# Bioactive Peptides Against Fungal Biofilms

**DOI:** 10.3389/fmicb.2019.02169

**Published:** 2019-10-04

**Authors:** Karen G. N. Oshiro, Gisele Rodrigues, Bruna Estéfani D. Monges, Marlon Henrique Cardoso, Octávio Luiz Franco

**Affiliations:** ^1^Programa de Pós-Graduação em Patologia Molecular, Faculdade de Medicina, Universidade de Brasília, Brasília, Brazil; ^2^S-Inova Biotech, Programa de Pós-Graduação em Biotecnologia, Universidade Católica Dom Bosco, Campo Grande, Brazil; ^3^Centro de Análises Proteômicas e Bioquímicas, Programa de Pós-Graduação em Ciências Genômicas e Biotecnologia, Universidade Católica de Brasília, Brasília, Brazil

**Keywords:** antifungal peptides, fungal infections, fungal biofilms, antimicrobial peptides, mechanisms of action

## Abstract

Infections caused by invasive fungal biofilms have been widely associated with high morbidity and mortality rates, mainly due to the advent of antibiotic resistance. Moreover, fungal biofilms impose an additional challenge, leading to multidrug resistance. This fact, along with the contamination of medical devices and the limited number of effective antifungal agents available on the market, demonstrates the importance of finding novel drug candidates targeting pathogenic fungal cells and biofilms. In this context, an alternative strategy is the use of antifungal peptides (AFPs) against fungal biofilms. AFPs are considered a group of bioactive molecules with broad-spectrum activities and multiple mechanisms of action that have been widely used as template molecules for drug design strategies aiming at greater specificity and biological efficacy. Among the AFP classes most studied in the context of fungal biofilms, defensins, cathelicidins and histatins have been described. AFPs can also act by preventing the formation of fungal biofilms and eradicating preformed biofilms through mechanisms associated with cell wall perturbation, inhibition of planktonic fungal cells’ adhesion onto surfaces, gene regulation and generation of reactive oxygen species (ROS). Thus, considering the critical scenario imposed by fungal biofilms and associated infections and the application of AFPs as a possible treatment, this review will focus on the most effective AFPs described to date, with a core focus on antibiofilm peptides, as well as their efficacy *in vivo*, application on surfaces and proposed mechanisms of action.

## Introduction

Fungal infections are recurrent in the clinical environment and, annually, affect ∼25% of the general population worldwide, causing high morbidity and mortality rates (Brown et al., 2012; Gamaletsou et al., 2018). The indiscriminate use of broad-spectrum antibiotics, along with parenteral nutrition, permanent catheters, chemotherapy and radiotherapy, as well as immunosuppression in patients, are the most important predisposing factors for invasive fungal infections (Lionakis and Levitz, 2018). Fungi are classified according to their morphologies, including yeasts (*Cryptococcus* spp.), fungi with branched hyphae (*Aspergillus* spp./*Rhizopus* spp.), as well as fungi with both morphologies (yeasts and pseudohyphas, as for *Candida* spp.), which have all been associated with fungal infections in humans (Brown et al., 2012; Lionakis and Levitz, 2018). In addition, fungal pathogens can also organize multicellular consortia, known as biofilms, which establish resistant communities on a variety of biotic and abiotic surfaces (Nett and Andes, 2015). Fungal adhesion to biotic and abiotic surfaces represents an initial stage by which fungi establish biofilms. Consequently, this cellular mechanism has been investigated as a potential target for antibiofilm therapies (Nett and Andes, 2015).

### Fungal Biofilms

Apart from their planktonic development, microorganisms can also establish biofilms in nature, and these biofilms allow microbial cells to survive in the host environment and be dispersed to colonize new niches (Hall-Stoodley et al., 2004). Fungal biofilms are composed of adherent cells covered by an extracellular matrix. First, free-floating cells adhere to a substrate followed by the secretion of an extracellular matrix, which confers additional mechanical protection on the fungal colonies. The release of biofilm cells is a regulated process by which organisms can spread throughout the host and establish new sites of infection (Nett et al., 2010; Uppuluri et al., 2010).

Studies have shown that biofilm-constituting cells usually present a different phenotype from that presented by planktonic cells. Among these differences, the elaborate architecture of biofilms has been highlighted as an additional challenge in the treatment of patients with systemic infections, mainly due to fungi’s increased resistance to conventional antibiotics and lower performance of the host immune system (Gulati and Nobile, 2016). Medical devices, including catheters and artificial heart valves, are in constant contact with body fluids, facilitating glycoprotein substrate deposition and favoring fungal cell adhesion, followed by their colonization and biofilm formation (Giles et al., 2018). Biofilm formation has been well described in *Candida albicans*, the most common fungal pathogen in the hospital setting (Douglas, 2002). *C. albicans* biofilms are composed of yeast and hyphalic cells, both of which are necessary for biofilm formation on biotic and abiotic surfaces (Dongari-Bagtzoglou et al., 2009; Harriott et al., 2010). Moreover, *Aspergillosis* and *Cryptococcus neoformans* biofilms are among the major causes of nosocomial infections caused by fungi (Ajesh and Sreejith, 2012; Kaur and Singh, 2013).

### Overview on Antifungal Peptides (AFPs)

Currently, antifungal therapies are scarce and include only four chemical classes of antifungal agents, namely polyenes, triazoles, echinocandins and flucytosine (Chowdhary et al., 2017). Moreover, the misuse of antifungal agents over the last two decades has contributed to antifungal resistance development (Perlin et al., 2017). Fungal resistance emergence has important clinical implications, as it limits the already small arsenal of antifungal agents, raising the idea of a “post-antifungal” era (Chowdhary et al., 2017). Therefore, an alternative is the use of AFPs against fungal infections and biofilms (Matejuk et al., 2010). AFPs have been tested as promising therapeutic agents for biofilm-related infections (Di Luca et al., 2014). In this context, AFPs have been considered a bioactive molecule group with broad-spectrum activities and multiple mechanisms of action. The search for AFPs capable of acting on fungal biofilms with lower toxic effects on mammalian cells either alone or in combination with conventional antibiotics has been the subject of diverse studies (Fjell et al., 2012). Although biofilm-active AFPs have not been achieved in clinical and commercial use, the development, design and optimization of such molecules remain as an alternative treatment (Duncan and O’Neil, 2013).

Antifungal peptides are structurally diverse. Moreover, AFPs comprise amphipathic molecules capable of interacting with biological membranes (De Lucca and Walsh, 1999; Faruck et al., 2016; Rautenbach et al., 2016). In the past decade, an increasing number of works have reported AFPs capable of either inhibiting fungal biofilm formation or eradicating preformed biofilms, as well as some AFPs with both inhibitory and eradication properties (Matejuk et al., 2010; Delattin et al., 2017). Most AFPs display their biological activities through membrane-associated mechanisms of action. When compared to other eukaryotic cells (e.g., mammalian cells), fungal membranes present few differences, including sphingolipid composition, PI content and the presence of ergosterol as the main sterol (Rautenbach et al., 2016). These differences, along with specific targets in the fungal pathogen, including fungal proteins, mannosyldiinositol phosphorylceramide and GlcCer, provide useful information for the generation of selective AFPs, avoiding toxicity toward human cells (Rautenbach et al., 2016). In this context, this review will focus on the therapeutic potential of AFPs, highlighting the most effective AFPs described to date, with a core focus on antibiofilm properties. Finally, we will explore the application of AFPs and their proposed antibiofilm mechanisms of action.

## Peptides With Deleterious Activity Toward Fungal Biofilms

### Defensin-Like Peptides

Defensins comprise AFPs isolated from various organisms, including plants and mammals (Cools et al., 2017a). Structurally, defensins are organized in an αβ motif, generally with an α-helix and a triple-stranded antiparallel β-sheet, which is stabilized by disulfide bonds that ensure high stability, thus retaining their functions under extreme conditions by avoiding/decreasing degradation (Shafee et al., 2016). Many defensins share the CSαβ motif, including plants, fungi and invertebrates. Therefore, the following subsections will address the structural characteristics, activity and mechanism of action of plant defensins, α-defensins and β-defensins on fungal biofilms.

#### Plant Defensins

**FIGURE 1:**
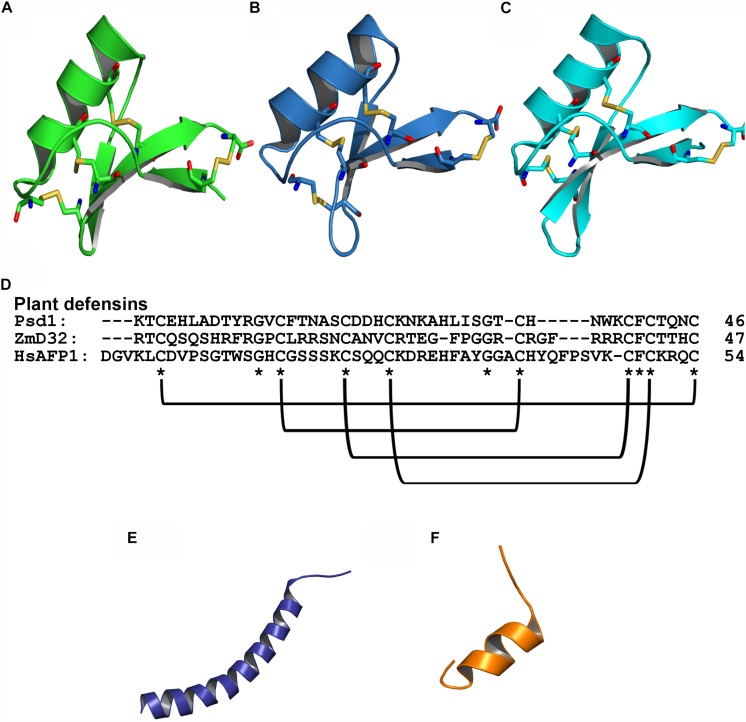
Tridimensional structure of AFPs with antibiofilm activity against fungi. **(A)** Psd1 – PDB: 1JKZ in green; **(B)** ZmD32 – PDB: 6DMZ in blue; **(C)** HsAFP1 – PDB: 2N2Q in cyan; **(D)** Sequence alignment of plant defensins showing the conserved regions and disulfide bonds; **(E)** LL-37 – PDB: 2K60 in purple; **(F)** BMAP-28 – PDB: 2NDC in orange; Disulfide bonds are highlighted as yellow sticks, oxygen atoms are in red and nitrogen atoms are in blue.

Plant defensins are cationic and have 45–54 amino acids in length. These peptides have typically been isolated from seeds, but can also be found in other plant tissues including leaves, flowers, roots and stems (Lay and Anderson, 2005). Most of the plant defensins identified so far have eight cysteine residues that favor structural stability by the formation of four disulfide bonds. In addition, structural studies have shown that plant defensins comprise a triple β-sheet with a parallel helix (Thomma et al., 2002) (Figure 1). Regarding their biological properties, plant defensins have shown activity against bacteria (Sathoff and Samac, 2019) and fungi, both in their planktonic and biofilm modes of growth (Vriens et al., 2016; Gonçalves et al., 2017).

**FIGURE 2:**
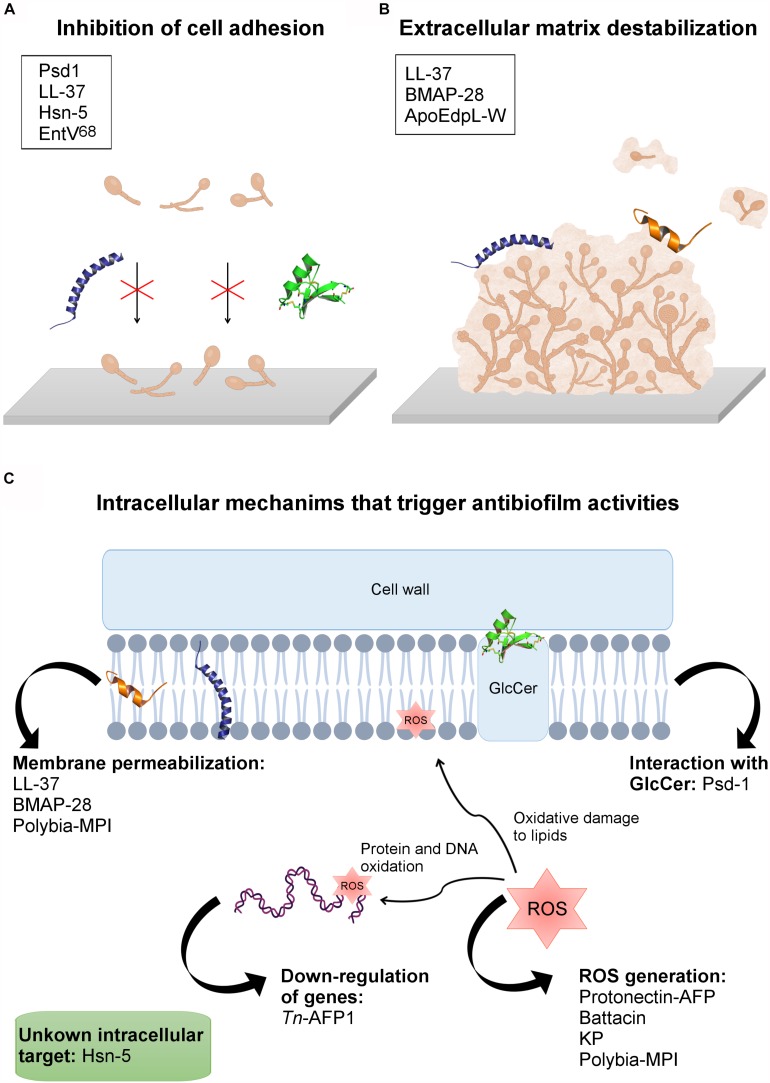
AFPs’ mechanisms of action against fungal biofilms. **(A)** Inhibition of cell adhesion. **(B)** AFPs’ interaction with the extracellular matrix, causing biofilm destabilization. **(C)** Peptides with intracellular mechanisms that trigger antibiofilm activities. In the boxes are the names of the peptides involved in each mechanism of action. The tridimensional structures present in the figure are: Psd1, in green (PDB: 1JKZ); LL-37 in purple (PDB: 2K60), and BMAP-28 in orange (PDB: 2NDC).

Psd1, for instance, is a plant defensin first isolated from *Pisum sativum seeds*, which has shown promising effects on *C. albicans* planktonic cells and biofilm (Gonçalves et al., 2017). Confocal microscopy and AFM analyses revealed that Psd1, at 20 μM, eradicates *C. albicans* planktonic cells; however, total inhibition or partial eradication of biofilms were observed at a concentration 10 x greater than the inhibitory value (approximately 200 μM) (Gonçalves et al., 2017). One of the main differences between mammalian and fungal cells is the presence of a cell wall in the latter. Thus, to access the fungal membrane, AFPs have first to interact with cell wall and plasma membrane components, which include sphingolipids, chitin, β-glucans and mannoproteins. The glycosphingolipid GlcCer, for instance, has been reported as a crucial plasma membrane component for anticandidal activity (planktonic cells and biofilms) of plant defensins (Aerts et al., 2008; Gonçalves et al., 2017). Psd1 acts on *C. albicans* biofilms by disaggregating the polysaccharide matrix of the cell wall (increasing cell roughness and decreasing its rigidity), followed by membrane permeabilization via interaction with GlcCer (Gonçalves et al., 2017). Once inside the fungal cells, Psd1 accumulation triggers an intracellular mechanism of action by interrupting the cell cycle, leading to apoptosis (Gonçalves et al., 2017). These mechanisms allowed Psd1 to decrease *C. albicans* planktonic cells’ adhesion, leading to the inhibition of biofilm formation, along with the eradication of preformed biofilms (Figures 2A,C).

In addition to Psd1, other plant defensins from *Heuchera sanguinea* coral bell seeds, named HsAFP1 and HsAFP2, have displayed deleterious antifungal activities against pathogenic fungi (Osborn et al., 1995; Aerts et al., 2011). Interestingly, HsAFP1 has been produced by heterologous expression using the yeast *Pichia pastoris*, and further denominated rHsAFP1 (r - recombinant). rHsAFP1 has shown inhibitory activity against planktonic *C. albicans* with a value for its MIC 50 (half maximal inhibitory concentration – 50%) of 18 μM. In addition, the minimal BIC50 was 11 μM (Vriens et al., 2015). In another study, 44 linear peptides derived from HsAFP1 were identified, among which the peptide HsLin06_18 showed antifungal activity (Cools et al., 2017b). This peptide (HsLin06_18) was evaluated against biofilms of *C. albicans*, obtaining a BIC value >2 μM. Interestingly, however, when in synergism with Csf this value (BIC) is reduced to 0.5 μM. In another study, a plant defensin isolated from *Raphanus sativus* antifungal protein 2 (RsAFP2) has also been expressed in *P. pastoris*. The recombinant peptide, rRsAFP2, has been proved to prevent *C. albicans* biofilm formation 330 μM (BIC). In contrary, rRsAFP2 is not capable of eradicating *C. albicans* biofilm (Vriens et al., 2016).

Plant defensins represent a large class of AFPs that exhibit remarkable stability at extreme pH and elevated temperature, as well as resistance to protease digestion (Kerenga et al., 2019). Kerenga et al. (2019) carried out a screening for plant defensin sequences in a database with more than 1,200 plant defensins, and identified a *Zea mays* defensin, named ZmD32, with the highest charge (+10) at pH 7. In this context, the objective of the work was to evaluate whether ZmD32 with an increased positive charge would retain the activity of the parent peptide at higher salt concentrations. It was observed that ZmD32 retained activity against a variety of fungal species in media containing high salt concentrations. In addition, ZmD32 has been shown to be multifunctional, as this peptide acted on fungi and on Gram-negative and Gram-positive bacteria. Moreover, the most prominent activity was observed against *C. albicans* biofilms, as ZmD32 eliminated biofilm viable cells from 20 to 40 μM at a physiological sodium concentration (132.1 mM). These findings demonstrate the potential of ZmD32 as a candidate for antifungal therapies (Kerenga et al., 2019).

#### α-Defensin

Defensins from vertebrate animals are cationic and amphipathic peptides containing 18–45 amino acid residues. These defensins have been divided into two subfamilies named α and β-defensins (Parisi et al., 2019). Human peptides have been isolated from neutrophils, playing important roles in processes related to host defense (Szyk et al., 2006; Jarczak et al., 2013). These peptides are cationic (arginine rich), with 3–5 kDa molecular masses, and they are stabilized by disulfide bonds (generally three) (Szyk et al., 2006). Human α-defensins have tertiary structure, and these defensins have a short helix structure and β-sheet stabilized by three disulfide bonds (Szyk et al., 2006).

An example of an α-defensin studied for its antifungal actions was human α-defensin 6 (HD6). This peptide demonstrated a blocking action of *C. albicans* adhesion in human intestinal epithelial cells, and treatment with HD6 at concentrations of 10 or 20 μM resulted in the prevention of biofilm formation (Chairatana et al., 2017). Some HBDs (e.g., HBD 2, 3, and 4) present antimicrobial properties and, in some cases (Krishnakumari et al., 2009) antifungal activities have been reported (Dhople et al., 2006; Krishnakumari et al., 2009). For instance, the α-defensin 6 (HD6) has demonstrated the potential to block *C. albicans* cells’ adhesion on human intestinal epithelial cells. Moreover, the treatment with HD6, at 10 or 20 μM, resulted in the prevention of biofilm formation (Chairatana et al., 2017).

#### β-Defensin

β-defensins comprise a large family of AMPs distributed in plants, mammals and insects. These cysteine-rich peptides are cationic and present approximately 45 amino acid residues (Taylor et al., 2008). The tertiary structures of β-defensins resemble α-defensin, the difference being in the position of the disulfide bonds responsible for stabilizing the β-sheet (Szyk et al., 2006; Shafee et al., 2016). Moreover, β-defensins have been reported for their multifunctional properties, including antifungal and antifungal biofilm activities (Taylor et al., 2008).

β-defensins comprise the majority of human defensins described with fungal antibiofilm activity. β-Human defensins (HBDs) are cationic peptides expressed in inflamed dental pulp (Paris et al., 2009). Similarly, different synthetic defensin-like peptides, including α-defensin-3, β-defensin-1, β-defensin-3 and PG-1, have demonstrated potent antifungal activity against *C. neoformans* biofilms. These peptides were effective against planktonic cells and mature biofilms, whereas PG-1, at 8 μM, reduced the survival percentage of cryptococcal biofilms by approximately 50%. β-defensin-1, at the same concentration, reduced biofilm survival by 60%, whereas α-defensin-3 and β-defensin-3 reduced biofilm survival by approximately 30% (Martinez and Casadevall, 2006). Interestingly, a 15-amino acid residue peptide derived from the C-terminus region of the human defensin 3 (HBD3-C15) has also revealed antifungal activity in a dose-dependent manner (28.3 – 169.8 μM) against *C. albicans* biofilm when evaluated on dentin disks (Lim et al., 2016).

The AFPs here cited are mainly active against *C. albicans* and *C. neoformans*. Plant defensins are stable AFPs with antifungal action and are capable of inhibiting biofilm formation, as well as eradicating preformed biofilms. In addition, these AFPs have also been proved to act in synergism with conventional antifungal agents, including HsLin06_18 peptide in combination with Csf, which acts on *C. albicans* biofilm formation (Vriens et al., 2016). Although these defensins have been proved to prevent fungal cell adhesion and, consequently, biofilm formation, more detailed studies regarding their antifungal biofilm activities are still scarce (van der Weerden et al., 2010). Therefore, further investigations with α-defensins are needed to demonstrate the real potential and mechanisms of action for this class of AFP. The same could be expected for β-defensins, which are active against fungal planktonic cells and biofilms, although the mechanisms by which they operate on different cellular organizations have not been yet elucidated. Thus, the findings here summarized, especially for plant defensins, represent an attractive field for further structure-function studies, aiming at correlating some of the already available tridimensional structures with AFPs’ mechanisms of action and, finally, identifying determinants for the generation of optimized AFPs that specifically target fungal biofilms. The AFPs described in the Defensin-like peptides section, their respective antibiofilm activities, and their proposed mechanisms of action are summarized in Table 1.

**TABLE 1:** Defensin-like peptides against pathogenic fungi and biofilms and their proposed mechanisms of action.

**Peptide**	**Source**	**Activity**	**Concentration (μM)**	**Pathogen**	**Mechanism of actions**	**References**
Psd1	*P. sativum* seeds	Biofilm inhibition	∼ 200	*C. albicans*	Cell cycle interruption, leading to apoptosis	Gonçalves et al., 2017
rHsAFP1	*H. sanguinea* coral bell seeds (r - recombinant)	Biofilm inhibition	BIC50^∗^ 11	*C. albicans*	Not determined	Vriens et al., 2015
rRsAFP2	*H. sanguinea* coral bell seeds (r - recombinant)	Biofilm inhibition	BIC^∗^ 330	*C. albicans*	Not determined	Vriens et al., 2016
HsLin06_18	Derived from rHsAFP1	Biofilm inhibition	BIC^∗^ > 2; and 0.5 synergism with Csf	*C. albicans*	Not determined	Cools et al., 2017b
ZmD32	*Z. mays*	Biofilm inhibition	20 – 40	*C. albicans*	Not determined	Kerenga et al., 2019
HD6	Human defensin	Biofilm inhibition	10 – 20	*C. albicans*	Not determined	Chairatana et al., 2017
β-defensin 1, β-defensin 3, PG-1	Human defensins	Biofilm inhibition	8	*C. neoformans*	Not determined	Martinez and Casadevall, 2006
HBD3-C15	Human defensin	Biofilm inhibition	Dose-dependent (28,3 – 169,8)	*C. albicans*	Not determined	Lim et al., 2016

*^∗^References values for each experiment, which can be: BIC, biofilm inhibition concentration; BIC50, concentration required to reduce biofilm formation by 50%.*

### Cathelicidins

Peptides from the cathelicidin family have been isolated from different species of mammals and exhibit broad-spectrum activities against fungi (Zanetti, 2004). Cathelicidins are characterized as cationic peptides, consisting of 12–80 amino acids that adopt an α-helix or β-sheet as secondary structures, most of which have 23–37 amino acid residues distributed in amphipathic helices, including LL-37 and BMAP-28 (Figure 1) (Zanetti et al., 1995; Gennaro and Zanetti, 2000).

The activities of LL-37 and BMAP-28 have been investigated against *Candida* spp. strains (clinical isolates of vaginal infections) in terms of planktonic cells’ growth inhibition and interference in fungal cell adhesion on polystyrene and silicone surfaces (biofilm formation) (Scarsini et al., 2015). LL-37, at 64 μM, was capable of inhibiting *C. albicans* cell adhesion on the tested surfaces (Scarsini et al., 2015). In that same work, BMAP-28, at 16 μM, was capable of inhibiting 70–90% of *C. albicans* and *Candida krusei* mature biofilms. BMAP-28 also reduced the number of *C. albicans* adherent cells on silicone surfaces, indicating its usage as an antifungal agent in coaching strategies (medical devices) (Scarsini et al., 2015). In addition to LL-37 and BMAP-18, a peptide derived from a cathelicidin-related AMP (CRAMP), named AS10, inhibited *C. albicans* biofilm formation at 0.22 μM (De Brucker et al., 2014). Furthermore, in synergism with Csf or amphotericin B, AS10 also acted on *C. albicans* mature biofilms (De Brucker et al., 2014). In that same work, another peptide, named P318, demonstrated even higher antifungal activity (0.15 μM) against *C. albicans* biofilms, without affecting planktonic cells’ survival (De Brucker et al., 2014).

The human cathelicidin LL-37 inhibits *C. albicans* adhesion and aggregation (2.2 and 4.5 μM) on biotic and abiotic surfaces by interacting with chitin, glucan and, especially, mannan present in the cell wall of this pathogen (Figure 2C) (Tsai et al., 2011). This ability has also been emphasized as a crucial mechanism by which LL-37 inhibits *C. albicans* biofilm formation on both medical devices and biological tissues in the course of *C. albicans* infections (Tsai et al., 2011). Moreover, to evaluate whether LL-37 interaction with mannan is selective for *C. albicans* or not, this AFP was also tested regarding its ability to interact with *Saccharomyces cerevisiae* mannan. As a result, LL-37 was not recovered by mannan from *S. cerevisiae* and, therefore, this AFP had no influence on cell aggregation and cell adhesion in this fungal strain (Figure 2A) (Tsai et al., 2011). It is known that the main difference between *C. albicans* and *S. cerevisiae* mannan is the presence of β-1,2 linkages in the first one (Shibata et al., 1985). This exclusive feature in *C. albicans* mannan was also proposed in that work (Tsai et al., 2011) as an important factor for LL-37 cell adhesion inhibitory potential, highlighting the application of this AFP in antibiofilm strategies.

Cathelicidins are peptides that are well described in the literature for their broad-spectrum activities and relatively stable structures. Cathelecidins have potential for the development of drugs that could be used on medical devices to combat fungal biofilms. Furthermore, the studies cited here demonstrate that this class can act synergistically with the conventional antifungal agents, rendering these peptides a promising starting point for future combinatorial therapies. The AFPs described in this section, their respective antibiofilm activities, and their proposed mechanisms of action are summarized in Table 2.

**TABLE 2:** Cathelicidin peptides against pathogenic fungi and biofilms and their proposed mechanisms of action.

**Peptide**	**Source**	**Activity**	**Concentration (μM)**	**Pathogen**	**Mechanism of actions**	**References**
LL-37	Human cathelicidin	Inhibited cell adhesion	64	*C. krusei* *C. albicans*	Membrane permeabilization	Scarsini et al., 2015
BMAP-28	Bovine cathelicidin	Biofilm inhibition - Biofilm eradication	16	*C. albicans*, *C. glabrata*, *C. krusei*	Membrane permeabilization	Scarsini et al., 2015
AS10 P318c	Derived from BMAP-18	Biofilm inhibition	0.22 0.15	*C. albicans*	Not determined	De Brucker et al., 2014
LL-37	Human cathelicidin	Biofilm inhibition	2.2 and 4.5	*C. albicans*	Adhesion and aggregation inhibition on biotic and abiotic surfaces	Tsai et al., 2011

### Histatins

Human salivary histatins are a group of small histidine-rich proteins constituted from 7 to 38 amino acids first isolated from human parotid saliva (Oppenheim et al., 1988). In general, histatins are a multifunctional group of proteins with antimicrobial properties that vary from broad-spectrum to moderate activities (Troxler et al., 1990). Moreover, histatins have been reported for their effective antifungal activity (Troxler et al., 1990; Tsai and Bobek, 1998). Histatins are divided into three major peptides, named histatin-1, histantin-3 and histatin-5, varying from 24 to 38 amino acid residues in length. They are polar and hydrophilic peptides presenting an α-helical structural conformation in organic solutions (Oppenheim et al., 1988). Therefore, histatin peptides have promising antifungal activities and their membrane affinity has been studied in the context of fungal infections (Brewer et al., 1998).

It is known that diverse pathogenic fungi can form biofilms on polymer surfaces, oral prostheses and medical devices. In that context, the activity of the Hst-5 peptide has been evaluated against *C. albicans* biofilms (CAI-4), demonstrating that this peptide, at 50 μM, inhibited biofilm formation on acrylic dentures *in vitro* (Pusateri et al., 2009). In another study, the potential of Hsn-5 was evaluated against planktonic cells and biofilms of *C. albicans* and *C. glabrata* growth on poly (methyl methacrylate) disks, simulating oral prostheses (Konopka et al., 2010). Hsn-5 was capable of inhibiting planktonic *C. albicans* strains with MICs ranging from 2.6 to 4.8 μM. In contrast, planktonic *C. glabrata* cells were insensitive to Hsn-5. Moreover, this peptide also caused a reduction in the biofilm metabolic activity (RMA) with concentrations ranging from 1.7 to 6.9 μM and from 31.2 and 62.5 μM against *C. albicans* and *C. glabrata* biofilms, respectively (Konopka et al., 2010).

Diverse studies have been carried out over recent decades aiming to understand the mechanism of histatin fungicidal activity. According to the literature, Hsn-5 acts by a multistep mechanism. First, the peptide is internalized by endocytosis, followed by its binding to the cell wall and further translocation across the membrane to act on intracellular targets (Figure 2C) (Sun et al., 2008). Hsn-5 can enter *C. albicans* by means of an energy-dependent or -independent mechanism (Moffa et al., 2015). Although we do not have complete evidence on the mechanisms of action of Hsn-5, studies have shown that the same peptide often has different mechanisms against planktonic cells and biofilms (Sun et al., 2008).

Some authors report that histidine-rich peptides, including the histatins cited in this section, are highly selective antifungals and have little toxicity toward mammalian cells. According to data here summarized, histatins demonstrate promising antifungal activities, making these AFPs potential candidates for biofilm treatment, especially oral fungal infections. However, although they have elucidated mechanisms of action against planktonic cells, histatin studies still lack deeper information on fungal antibiofilm properties and mechanisms of action, making this class a potential subject for further studies aimed at combating fungal biofilms, determining their tridimensional structures, as well as unraveling their mechanisms of action. The AFPs described in this section, their respective antibiofilm activities, and their proposed mechanisms of action are summarized in Table 3.

**TABLE 3:** Histatin peptides against pathogenic fungi and biofilms and their proposed mechanisms of action.

**Peptide**	**Source**	**Activity**	**Concentration (μM)**	**Pathogen**	**Mechanism of actions**	**References**
Hst-5	Human salivary histatins	Biofilm inhibition	50	*C. albicans*	Not determined	Pusateri et al., 2009
Hsn-5	Human salivary histatins	Biofilm inhibition	1.7 – 6.9 4.8 31.2 – 62.5	*C. albicans* *C. glabrata*	Peptide is internalized by endocytosis, then it binds to the cell wall and translocates into the cell to act on intracellular targets	Sun et al., 2008; Konopka et al., 2010

### Miscellaneous AFPs That Act on Fungal Biofilms

In addition to the above-mentioned classes of AFPs with antibiofilm potential, studies have also described additional AFPs from different sources that target fungal cell adhesion, thus inhibiting biofilm formation, as well as preformed biofilms. Ergosterol is the main sterol that constitutes fungal membranes. Moreover, studies have shown that the overexpression of genes involved in the biosynthesis of ergosterol (e.g., *ERG11*, *ERG16* and *ERG25*) may be crucial for *Candida* species biofilm formation (Garcia-Sanchez et al., 2004). For instance, the peptide *Tn*-AFP1, which is derived from *Trapa natans*, demonstrated antifungal potential by inhibiting planktonic cells of *C. tropicalis* at 26 μM. Moreover, it has been reported that *Tn*-AFP1 is capable of inhibiting fungal biofilm formation in a dose-dependent manner, as well as eradicating preformed biofilms (Mandal et al., 2011). In addition, when evaluating the levels of expression of two genes related to biofilm formation, including *ERG11* (ergosterol biosynthesis) and *MD1R* (ATP-binding cassettes pump) it was observed that planktonic cells treated with *Tn*-AFP1 presented the down-regulation of these genes and, therefore, could not establish biofilms (Figure 2C) (Mandal et al., 2011). Moreover, this AFP also induced morphological cell changes, along with fungicidal effects on biofilm-constituting cells.

In addition, a decapeptide isolated from *Arabidopsis thaliana*, called OSIP108, was evaluated regarding its antifungal and antibiofilm properties on *C. albicans* (De Coninck et al., 2013). As a result, the authors observed that OSIP108, from 2 to >200 μM, did not display antifungal activity against *C. albicans* planktonic cells, whereas OSIP108, from 6.25 to 100 μM, reduced *C. albicans* biofilm formation when administrated during the cell adhesion stage. These findings indicate that, despite using the same AFP, its antifungal and antibiofilm modes of action are most likely to be independent, as AFPs that are promising against planktonic cells may present ineffective antibiofilm properties and vice versa.

As described above, human-derived peptides have also been pinpointed as promising antifungal agents. A tryptophan-rich peptide derived from the human ApoE apolipoprotein (ApoEdpL-W), for instance, has shown antifungal activity against pathogenic yeasts of the *Candida* species, except for *C. glabrata* (Rossignol et al., 2011). ApoEdpL-W was active against planktonic cells and biofilms at early stages, but less active against mature biofilms (10 to 80 μM). In addition, ApoEdpL-W partially prevented biofilm formation on medical devices (Rossignol et al., 2011). Fungal cells in biofilms are embedded in an extracellular matrix composed of exopolymeric compounds, including β-1,3 glucan. Taking that into account, Rodrigues et al. (2018) evaluated the susceptibility of *C. glabrata* biofilms to echinocandins (cyclic lipo-hexapeptides), including Csf and micafugin (Mcf), also shedding some light on how these two AFPs interfere in β-1,3 glucan concentration in the matrix of *C. glabrata* biofilms. The authors observed that *C. glabrata* preformed biofilms treated with Csf and Mcf presented adjustments in the matrix composition due to a decrease in β-1,3 glucan concentration (Rodrigues et al., 2018). These findings were further correlated with the antibiofilm potential of these two AFPs, partially elucidating their antibiofilm mechanism (Rodrigues et al., 2018). A similar hypothesis has also been proposed for the higher antibiofilm activity of the tryptophan-rich AFP, ApoEdpL-W, against early-stage *C. albicans* biofilms when compared to mature biofilms (Rossignol et al., 2011). This finding can be partially explained by the affinity of ApoEdpL-W for extracellular matrix β-glucans in mature biofilms (e.g., β-1,3 glucan), which is known to trap antifungal agents and, consequently, confer biofilm tolerance (Figure 2B) (Rossignol et al., 2011).

Insect peptides are also known for their broad-spectrum of biological activities. Polybia-MPI, for instance, was originally isolated from the venom of the social wasp *Polybia paulista* and presented potent antibacterial activity (Souza et al., 2005). To better understand peptide biological potential, Polybia-MPI was evaluated for its inhibitory, fungicidal and antibiofilm activities against *Candida* spp. (Wang et al., 2014). Polybia-MPI revealed MIC and MFC values of 16 and 32 μM against *C. albicans*, respectively, whereas *C. glabrata* was inhibited (MIC) and killed (MFC) by Polybia-MPI at 8 and 32 μM, respectively. In addition, Polybia-MPI inhibited *C. glabrata* biofilm formation on polystyrene surface from 2 × MIC to 8 × MIC, resulting in a drastic decrease of biofilm biomass (Wang et al., 2014).

Another example of an AFP isolated from wasp toxin is the protonectin peptide, which was originally isolated from *Agelaia pallipes pallipes* (Mendes et al., 2004). Protonectin has been evaluated against *C. glabrata*, *C. albicans*, *C. parapsilosis*, *C. tropicalis* and *C. krusei*, revealing MICs from 8 to 128 μM. Protonectin was also found to disrupt fungal membrane integrity and induce the production of cellular reactive oxygen species (ROS), inhibiting the formation of *C. glabrata* biofilms (Wang et al., 2015). Wang et al. (2015), for instance, observed that a protonectin AFP, derived from the venom of a social wasp, has potent antifungal and fungicidal activities. Moreover, this AFP not only inhibited biofilm formation, but also killed adherent biofilm cells. All these activities were further correlated with membrane-associated mechanisms, along with the generation of ROS (Figure 2C) (Wang et al., 2015). In fungi, ROS are generated as metabolic products from an endogenous or exogenous source and include hydrogen peroxide and hydroxyl radicals, which act as signaling molecules for gene regulation (Scandalios, 2002; Cho and Lee, 2011). Inside fungal cells, ROS generation is balanced by the production of antioxidants. However, when this balance is compromised (for instance, by the presence of AFPs) ROS accumulation may lead to oxidative damage to lipids, proteins and DNA, resulting in cell death (Scandalios, 2002; Cho and Lee, 2011). In antifungal therapies focusing on AFPs, antifungal properties toward fungal biofilms have been reported and, in some cases, associated with ROS generation. Similar findings were reported for linear battacin peptides against *C. albicans* mature biofilms (Figure 2C) (De Zoysa et al., 2018).

Peptides derived from insect venom have also been submitted to sequence optimization strategies aiming at improved antifungal properties. Lasioglossin-III (LL-III) and halitin (HAL-2), for instance, represent two peptides derived from bee venom (Čeřovský et al., 2009) that were used as template molecules for the generation of synthetic analogs, named LL-III/43 and peptide VIII, respectively. These analogs were evaluated for their antifungal and antibiofilm activities against *Candida* spp. The lowest MIC (0.8 μM) value was observed for LL-III/43 against *C. tropicalis*. Moreover, both analogs inhibited *Candida* spp. biofilm formation, with concentrations ranging from 0.9 to 58.6 μM. Biofilm eradication for almost all *Candida* species tested ranged from 12.8 to 200 μM (Kočendová et al., 2019).

Bacteriocins are examples of peptides produced by various bacterial species with antifungal action (Sang and Blecha, 2008). EntV is a bacteriocin encoded by the entV locus (ef1097) from *Enterococcus faecalis* (Graham et al., 2017), originally studied for its antibacterial activity against Gram-positive strains (Swe et al., 2007). Graham et al. (2017) have reported a synthetic bacteriocin version, named EntV^68^, which is constituted of 68 amino acid residues with one disulfide bond involved in structure cyclization. EntV^68^ has been shown to be effective for reducing virulence of *C. albicans* strains and biofilm formation by inhibiting hyphae formation (BIC50 = 0.0003 μM) (Figure 2A). Furthermore, this peptide potentially blocks biofilm development in solid substrates under multi-media conditions and has been proved to disturb preformed biofilms that are resistant to current antifungal agents (Graham et al., 2017). In addition, that work also evaluates whether EntV^68^ protected phagocytes from *C. albicans*-induced damage or not (Graham et al., 2017). The murine RAW 264.7 macrophages were incubated with *C. albicans* cells in the presence and absence of EntV^68^, at 100 nM (0.0001 μM). Under the peptide presence, the authors observed a decrease in the release of lactate dehydrogenase (LDH), an enzyme present in the macrophage membrane. Moreover, the analysis showed fewer surviving fungal cells, indicating that EntV^68^ reduces fungus-induced cytotoxicity and may potentiate macrophage antifungal activity (Graham et al., 2017). Therefore, EntV^68^ has clear pharmacological potential, as this peptide is capable of inhibiting biofilm formation and also disturbing preformed biofilms, without causing cytotoxicity in macrophages. In addition, two *in vivo* experiments were performed, which will be described later in the section “AFPs used to counter fungal infections in animal models.”

In addition to insects, other arthropods and also bacteria can be a rich source of bioactive peptide screening. Lichosin-1, which is derived from *Lycosa singoriensi* spider venom (Tan et al., 2018), showed antifungal activity against clinical isolates of fluconazole-resistant *C. albicans*, with MIC values from 0.31 and 0.67 μM. When this peptide was evaluated against *C. albicans* biofilm, it was capable of inhibiting biofilm formation from 2.75 to 70.73 μM. However, higher concentrations ranging from 136.25 to 694.47 μM were required for antibiofilm activities against mature biofilms (Tan et al., 2018). In addition, Lycosin-1 acts against *C. tropicalis* through several types of morphological damage, leading to decreased biofilm filamentation, along with an increased number of gaps between cell clusters within the biofilms (Tan et al., 2018).

Another study evaluated optimized synthetic peptides called kaxins, capable of inhibiting fluconazole-susceptible and –resistant *C. albicans*, *C. tropicalis* and *C. glabrata* strains (128 to 512 μM) (Burrows et al., 2006). In that work, it was observed that a kaxin peptide, named dF21-10K, completely eradicated *C. albicans* and *C. tropicalis* preformed biofilms in a concentration 10-fold higher than the MIC against these strains (61.5 to 246.1 μM) (Burrows et al., 2006).

Also in the field of synthetic peptides, a decapeptide known as killer peptide (KP) was described (Paulone et al., 2017). This peptide was tested against fluconazole-resistant and -susceptible *C. albicans* biofilms at different development stages (cell adhesion, development of hyphae and extracellular matrix production). KP exerted fungicidal activity against all planktonic strains investigated, with MFC from 0.31 to 0.67 μM. The inhibitory effects of KP in *C. albicans* biofilm’s early stages showed that 124.2 μM, KP impaired the biofilms, reducing the total biomass by more than 45% in four strains. Furthermore, the inhibitory effects of KP on mature (2-day old) biofilms of *C. albicans* showed that, at 124.2 μM, KP significantly impaired the total biomass of mature biofilms. In addition, KP administration led to an increased oxidative stress response in *C. albicans*, showing that this peptide has inhibitory effects on *C. albicans* biofilm regardless of whether this pathogen is resistant or susceptible to fluconazole (Paulone et al., 2017). AFPs can present multiple mechanisms against fungal biofilms depending on their stage, including early and mature biofilms. Recently, it was reported that a synthetic killer decapeptide (KP) was capable of inhibiting fluconazole-susceptible and -resistant *C. albicans* biofilm formation, also significantly affecting the viability of preformed biofilms (Paulone et al., 2017). In that work, KP induced ROS production in mature biofilms and also decreased the viability of biofilm-constituting fungal cells through membrane permeabilization (Paulone et al., 2017). Moreover, the transcriptional profile of *C. albicans* biofilms in early and mature stages treated with KP indicates that this AFP reduced the expression of biofilm-associated genes, including matrix-related genes and hyphal-specific genes (Figure 2C) (Rodrigues et al., 2018).

Battacin, a cyclic lipopeptide isolated from *Paenibacillus tianmuensis*, has shown promising antibacterial activities (Qian et al., 2012). De Zoysa et al. (2015) developed 16 battacin linear analogs, observing an improvement in their activity against bacterial biofilms. Based on that, these analogs were also submitted to fungal antibiofilm assays. As a result, the authors identified the three most effective analogs (3, 12, and 13), which were capable of inhibiting planktonic cells of *C. albicans* at 50, 12.5, and 6.25 μM, respectively. Since the lowest inhibitory concentration was observed for analog 13, it was evaluated against *C. albicans* biofilms. Analog 13 showed BIC50 values of 6.25 μM and was able to eradicate pre-formed biofilms 62.5 μM (10 times its MIC) (De Zoysa et al., 2018). The AFPs described in this section, their respective antibiofilm activities, and their proposed mechanisms of action are summarized in Table 4.

**TABLE 4:** Unusual AFP classes of peptides against pathogenic fungi and biofilms and their proposed mechanisms of action.

**Peptide**	**Source**	**Activity**	**Concentration (μM)**	**Pathogen**	**Mechanism of actions**	**References**
*Tn*-AFP1	*T. natans*	Antifungal- Biofilm inhibition - Biofilm eradication	26 (inhibition) 52 (eradication)	*C. tropicalis*	Down-regulation of genes (*ERG11* and *MD1R*) and, therefore, cannot establish biofilms	Mandal et al., 2011
dF21-10K	Synthetic peptide	Biofilm eradication	61.5 – 246.1	*C. albicans* *C. tropicalis*	Not determined	Burrows et al., 2006
OSIP108	*A. thaliana*	Biofilm inhibition	6.25 – 100	*C. albicans*	Not determined	Delattin et al., 2014
ApoEdpl-W	Human ApoE apolipoprotein	Biofilm inhibition	10 – 80	*Candida* spp., except for *C. glabrata*	Affinity for extracellular matrix β-glucans in mature biofilms, conferring biofilm tolerance	Rossignol et al., 2011
Polybia-MPI	*P. paulista*	Biofilm inhibition	16 – 32 8 – 32	*C. albicans*, *C. glabrata*	Generation of ROS	Wang et al., 2014
LL-III/43 Peptide VIII	Bee venom	Biofilm inhibition - Biofilm eradication	0.9 – 58.6 (inhibition) 12.8 – 200 (eradication)	*C. tropicalis Candida* spp.	Not determined	Kočendová et al., 2019
KP	Synthetic peptide	Biofilm inhibition Biofilm eradication	0.31 – 0.67 124.2	*C. albicans*	ROS generation in mature biofilms and membrane permeabilization	Paulone et al., 2017
Protonectin AFP	*A pallipes pallipes*	Biofilm inhibition - Biofilm eradication	–	*C. albicans*	ROS generation	Wang et al., 2015
Battacin	*P. tianmuensis*	Biofilm inhibition - Biofilm eradication	BIC50^∗^ 6.25 (inhibition) 62.5 (eradication)	*C. albicans*	ROS generation	De Zoysa et al., 2018
Lichosin-1	*L. singoriensi*	Biofilm inhibition - Biofilm eradication	2.75 – 70.73	*C. albicans*	Not determined	Tan et al., 2018
EntV^68^	bacteriocin of *E. faecalis*	Biofilm inhibition - blocking biofilm development	BIC50^∗^ 0.0003 0.0001	*C. albicans* (virulence and biofilm development) *C. albicans, C. tropicalis, C. parapsilosis C. glabrata* (inhibition of formed biofilms)	Reduces virulence of *C. albicans* strains and biofilm formation by inhibiting hyphae formation blocking biofilm development in solid substrates under multi-media conditions	Graham et al., 2017

*^∗^Reference values for each experiment, which can be: BIC50, concentration required to reduce biofilm formation by 50%.*

## AFPs Used to Counter Fungal Infections in Animal Models

In general, antifungal and antibiofilm peptides are tested in murine models, although different models have been developed in primates, rabbits, guinea pigs, birds, and canines (Hohl, 2014; Dijck et al., 2018). Animal models are an effective method by which to evaluate the progression of fungal pathogenesis and host immune responses and to investigate the antifungal properties of drug candidates (Capilla et al., 2007; Hohl, 2014; Dijck et al., 2018). These models allow the control of different biological variables, mimicking human diseases and monitoring disease progress (Capilla et al., 2007; Dijck et al., 2018). Recently, studies have used non-vertebrate models to optimize the screening of candidate drugs and evaluate fungal virulence. Non-vertebrate and other animal models are described in Table 5. According to Segal and Frenkel (2018), animal models are classified based on methods of therapeutic evaluation, including superficial (skin, nails), mucosal (oral, vaginal), gastrointestinal and lung or systemic infections (intravenous, intraperitoneal) (Capilla et al., 2007; Hohl, 2014; Dijck et al., 2018). In addition, antifungal drugs have limited efficiency against invasive fungal infections, directly impacting increasing mortality rates (Hohl, 2014).

**TABLE 5:** Overview of different animal models for screening for antifungal drugs.

**Animal models**	***Candida* sp.**	***Aspergillus* sp.**	***Cryotococcus* sp.**
*Galleria mellonella* (greater wax moth)	Rowan et al., 2009; Lopez-Moya et al., 2015; Souza et al., 2015; Aneja et al., 2016; Ames et al., 2017; Gu et al., 2018; Lu et al., 2018	Alcazar-Fuoli et al., 2015; Forastiero et al., 2015; Maurer et al., 2015; Ben Yaakov et al., 2016; Ben Yaakov et al., 2017	Sangalli-Leite et al., 2016; Palanco et al., 2017; de Sá et al., 2018
*Bombyx mori* (silkworm)	Uchida et al., 2016	Nakamura et al., 2017	Ishii et al., 2016; Matsumoto et al., 2017
*Caenorhabditis elegans*	Delattin et al., 2013; Muhammed et al., 2016; Graham et al., 2017; Mohammad et al., 2018; Subramenium et al., 2018; Sun et al., 2018		de Aguiar Cordeiro et al., 2016; Thangamani et al., 2017
*Drosophila melanogaster*	Glittenberg et al., 2011; Zanette and Kontoyiannis, 2013	Lionakis and Kontoyiannis, 2012	
*Danio rerio* Zebrafish larvae			Palanco et al., 2017
Mice	López-García et al., 2005; Graham et al., 2017; Li et al., 2017; Peters et al., 2017; Wu et al., 2017; Dostert et al., 2018; Singulani et al., 2017; Lepak et al., 2018; Ci et al., 2018	Ben-Ami et al., 2010; Kai et al., 2013; Paulussen et al., 2015	Rathore et al., 2017; Nixon et al., 2018
Rats	Bink et al., 2012; Kucharíková et al., 2013; De Cremer et al., 2014; Kucharíková et al., 2014; Li et al., 2014; Cools et al., 2017b; Holtappels et al., 2018		
Guinea pigs	Maiolo et al., 2016	Wiederhold et al., 2015; Zhao et al., 2015	Kirkpatrick et al., 2007
Rabbit		Walsh et al., 1995; Petraitiene et al., 2002	

In this context, studies with animal models demonstrate a reliable strategy for evaluating the effectiveness of AFPs on biofilm-associated fungal infections, thus helping researchers to elucidate the therapeutic application of these antifungal agents in the clinic. Therefore, here we described different AFPs with activity against fungal biofilm in distinct animal models. Cools et al. (2017b) tested HsLin06_18 (derived from the plant defensin HsAFP1) in association with Csf. This combination was tested in immunosuppressed female Sprague–Dawley rats. *C. albicans* biofilms were formed inside catheters (5 × 10^4^ cells. mL^–1^), which were further implanted into the back area of the rats. The antibiofilm treatment aiming to inhibit biofilm formation was initiated immediately after the implant. The combination Csf + HsLin06_18 and the control group were administered intravenously or subcutaneously once daily for 7 days. It was demonstrated that the combination Csf + HsLin06_18 reduced *C. albicans* biofilm formation *in vivo* compared to the untreated control, besides not presenting cytotoxicity in healthy cells. Similar effects have been observed for an insect defensin, named drosomycin, against *Botrytis cinerea* strain B05-10 and *Colletotrichum gloeosporioides.* Drosomycin inhibited the growth of both fungi at 1.5 and 15 μM, respectively (Cohen et al., 2009). Another group demonstrated the efficiency of AFPs to combat planktonic fungi and biofilm formation. In a study using OSIP108, a *Caenorhabditis elegans in vivo* model was used to test synergic effects using different combinations of OSIP108 with Csf. The worms were infected with *C. albicans* and subsequently treated with 100 μM OSIP108, 0.095 μM Csf, and 100 μM OSIP108 + 0.095 μM Csf, and 0.6% DMSO (negative control) after 3, 5, and 7 days. The authors reported that OSIP108 alone did not present activity in *C. albicans* infected worms. However, the combination 100 μM OSIP108 + 0.095 μM Csf improved worms’ survival. In accordance with these results, OSIP108 can be used for coating strategies in some medical devices, assisting in the fight against biofilm formation. The studies described above show that AFPs derived from plants are very efficient in inhibiting or controlling fungal biofilms tested in different *in vivo* models, demonstrating their therapeutic potential (Thevissen et al., 2007; Delattin et al., 2014).

Different groups of peptides have been tested by Yu et al. (2016). These authors have demonstrated the potential of four cathelicidins (cathelicidin-BF, Pc-CATH1, Cc-CATH2, Cc-CATH3) in combating *C. albicans* biofilm formation (Yu et al., 2016). The cathelicidins were tested in a murine oral candidiasis model using LL-37 and amphotericin B as control. Mice were infected by intramuscular injection and then infected by topical inoculation with *C. albicans* dilutions (0.1 mL) on the oral mucosa surface. The cathelicidin-BF inhibited *C. albicans* biofilm formation, demonstrating better results compared to other peptides. Similar results were also described by De Brucker et al. (2014) in *in vitro* tests using AS10 to inhibit fungal biofilm formation. As a result, AS10 was capable of inhibiting biofilm formation at 0.22 μM, and acted synergistically with amphotericin B and Csf against mature biofilms. In addition, this peptide did not exert a cytotoxic effect on mammalian cells.

Furthermore, synthetic peptides (β-peptides) have shown promising antifungal and antibiofilm properties against *C. albicans.* These AFPs act by reducing fungal metabolic activities and preventing or compromising biofilm formation (Delattin et al., 2014; Raman et al., 2014). The great activity of synthetic β-sheet peptides was also demonstrated by Wu et al. (2015). These authors tested the peptides (IKIK)_2_-NH_2_ and (IRIK)_2_-NH_2_ *in vivo* against fungal keratitis in comparison with the commercially available amphotericin B (Wu et al., 2015). They used contact lenses containing a layer of *C. albicans* biofilm, subsequently transferred onto the de-epithelia cornea surface of mice. The inoculum was maintained for 18 h, and eye ulcers developed with a leathery, tough, raised surface. Treatment was performed with peptide 1 (3000 mg.L^–1^), peptide 2 (3000 mg.L^–1^), amphotericin B (1000 mg.L^–1^) and water (control), and further applied topically as eye drops (20 mL) on the corneal surface. After treatment, the authors observed a significant decrease in keratitis infection, suggesting that synthetic β-sheet peptides are effective in removing keratitis-related fungal biofilms from mouse eyes.

Additionally to the previously mentioned *in vitro* experiments, the potential of EntV^68^ was evaluated in two fungal infection models in which this peptide showed to be protective during *C. albicans* infection via inhibition of hyphal morphogenesis at low concentrations. The nematode infection model used *C. elegans* to evaluate *C. albicans* filamentation by microscopy. Thus, it was observed that, in the presence of EntV^68^ at subnanomolar concentrations (100 nM = 0.0001 μM), the virulence of *C. albicans* in the nematodes was nullified. Furthermore, the authors suggest that EntV^68^ is effective in protecting *C. elegans* during *C. albicans* infection via inhibition of *C. albicans* hyphal morphogenesis (Graham et al., 2017). In murines (immunosuppressed Balb-C mice), the evaluated model was oropharyngeal candidiasis (OPC). The results obtained showed that, based on a treatment with 100 nM (0.0001 μM) of EntV^68^, mice had a significant reduction in fungal cell invasion, showing once again the ability of this peptide to inhibit morphological differentiation (Graham et al., 2017). Other peptides tested in animal models against fungal strains and biofilms are summarized in Table 6.

**TABLE 6:** Overview of antifungal peptides tested *in vivo* against free-floating fungi and biofilms.

**Peptides**	**AFP classes**	**References**
hBD1hDB3, Psd1, HsAFP1, RsAFP2, NFAP2,	Defensins	Martinez and Casadevall, 2006; Pusateri et al., 2009; Delattin et al., 2014; Gonçalves et al., 2017; Menzel et al., 2017; Kovács et al., 2018
LL-37, P318, AS10	Cathelicidins	Benincasa et al., 2006; De Brucker et al., 2014; Yu et al., 2016; Niemirowicz et al., 2017; Sun et al., 2018
Histatin-5, Hst5_4__–__15_Spd	Histatins	Sung et al., 2008; Tati et al., 2014
*Tn*-AFP, OSIPI08, EntV^68^	Unusual AFPs	Gonçalves et al., 2017; Kovács et al., 2018
β-peptides, mPE	Synthetic β-sheet	Raman et al., 2014; Wu et al., 2015

## Biotechnological Potential

In recent years, fungal infections have become a worldwide health problem (Brown et al., 2012; Gamaletsou et al., 2018). Fungal biofilm formation is nowadays increasingly reported in systemic, superficial and mucosal fungal infections (Duncan and O’Neil, 2013; Nett and Andes, 2015). In addition to that, therapeutic strategies are still scarce and show limited effectiveness (Chowdhary et al., 2017; Santos et al., 2017). Moreover, one of the ways to correlate the challenges of working with AFPs is by looking at the failures of working with AMPs. In both cases, one of the obstacles in developing such molecules as pharmaceuticals is the substantial activity loss under physiological saline concentrations (Kerenga et al., 2019). Some authors have reported additional disadvantages, including systemic and local toxicity, susceptibility to proteolysis, sensitization and allergy after repeated application, and high costs in discovering, screening and manufacturing these peptides (Koczulla and Bals, 2003; Gordon et al., 2005). However, there are promising advantages as well, including AFPs’ broad-spectrum action (antibacterial, antifungal, antiviral), rapid action upon contact with the pathogen, potentially low levels of induced resistance, as well as anti-inflammatory and immunomodulatory activity (Koczulla and Bals, 2003; Gordon et al., 2005). After years without any innovation in chemical antifungal agents, the AFPs initiated a new prospect for fungal treatment (Duncan and O’Neil, 2013; Santos et al., 2017). The study of AFPs is an emerging field, and recent works have focused on new strategies to improve their stability, safety, and efficacy (Kerenga et al., 2019). Moreover, an increasing number of studies have shown the advantages of combination therapies and drug delivery systems for AFPs’ efficacy (Mahlapuu et al., 2016; Koo et al., 2017; Gomes et al., 2018; Revie et al., 2018).

According to the Food and Drug Administration (FDA), the number of approved bioactive peptides is growing. This rise is associated with the understanding of biofilm microenvironments, allowing the development of multi-targeted therapeutic approaches to prevent biofilm formation and combat preformed biofilms, enhancing drug efficacy (Koo et al., 2017; Lee et al., 2019). Some AFPs have been proved to act synergically with conventional antifungals, improving the success of antifungal therapies (Cohen et al., 2009; De Brucker et al., 2014). Thus, AFPs have shown great efficiency against fungal biofilms, along with the lack of side effects that are recurrent in conventional antifungal treatments. Moreover, some AMPs are undergoing pre-clinical and clinical trials, including the treatment of infections related to contaminated catheters, topical formulations for acne, treatment of peritoneal infections caused by bacteria (e.g., pneumonia), as well as treatment of gingivitis and oral biofilms (Guaní-Guerra et al., 2010). However, to date, AFPs with fungal antibiofilm activity have not yet reached the market.

Therefore, in this review we described different applications for AFPs, with a core focus on antibiofilm properties toward fungi. This includes the use of AFPs as topical agents for the treatment of superficial vulvovaginal candidiasis (Kovács et al., 2018). Another application is against fungal keratitis, in which AFPs can be used as eye drops (Wu et al., 2015), as well as for oral administration (Yu et al., 2016). Furthermore, AFPs can be immobilized on medical devices (e.g., catheter, prosthesis and implants) to prevent fungal biofilm formation (De Brucker et al., 2014; Gonçalves et al., 2017). Besides that, nanoformulation strategies may allow the maintenance of AFPs stability and activity, thus improving the treatment’s effectiveness by creating drug delivery systems (Batoni et al., 2011; Kovalainen et al., 2015; Mahlapuu et al., 2016; Cavalheiro and Teixeira, 2018; Dostert et al., 2018). Apart from their strong performance in the therapeutic area, AFPs can also be used in cosmetics (Bedoux et al., 2014; Rahnamaeian and Vilcinskas, 2015; Carvalho et al., 2016), diagnostics (Ribeiro et al., 2016; Young-Speirs et al., 2018), functional food and nutraceuticals (Gianfranceschi et al., 2018), vaccines (Nami et al., 2019), and in agriculture for pest control (Subbanna et al., 2019).

When combined with their activity against fungal biofilms, heat stability, pH, degradation and proteolysis, we can provide a solid basis for the development of AFPs as antimicrobial therapeutic agents for clinical use. Their multifunctionality with respect to antifungal and antibacterial properties is particularly stimulating, as there is potential utility against polymicrobial infections. Taken together, all the findings highlighted in this review suggest the promising application of AFPs as new biomolecules in pre-clinical and clinical trials, reinforcing a growing movement in which bioactive peptides may assume a lead role in modern medicine and pharmaceutics.

## Author Contributions

KO, GR, BM, and MC wrote the manuscript. KO and MC idealized and organized the figures. MC and OF corrected the manuscript. OF supervised and managed all authors.

## Conflict of Interest

The authors declare that the research was conducted in the absence of any commercial or financial relationships that could be construed as a potential conflict of interest.

## Abbreviations

AFM: atomic force microscopy
AFPs: antifungal peptides
AMPs: antimicrobial peptides
BIC: biofilm inhibition concentration
BIC50: concentration required to reduce biofilm formation by 50%
Csf: caspofungin
GlcCer: glucosylceramide
Mcf: micafungin
MFC: minimal fungicidal concentration
MIC: minimal inhibitory concentration
PI: phosphatidylinositol.
